# Modeling and performance analysis dataset of a CIGS solar cell with ZnS buffer layer

**DOI:** 10.1016/j.dib.2017.07.054

**Published:** 2017-07-26

**Authors:** Md. Billal Hosen, Ali Newaz Bahar, Md. Karamot Ali, Md. Asaduzzaman,

**Affiliations:** Department of Information and Communication Technology (ICT), Mawlana Bhashani Science and Technology University (MBSTU), Tangail 1902, Bangladesh

**Keywords:** Numerical dataset, CIGS, Solar cell simulation, ZnS buffer, Conversion efficiency

## Abstract

This article represents the baseline data of the several semiconductor materials used in the model of a CIGS thin film solar cell with an inclusion of ZnS buffer layer. As well, input parameters, contact layer data and operating conditions for CIGS solar cell simulation with ZnS buffer layer have been described. The schematic diagram of photovoltaic solar cell has been depicted. Moreover, the most important performance measurement graph, J-V characteristic curve, resulting from CIGS solar cell simulation has been analyzed to estimate the optimum values of fill factor and cell efficiency. These optimum results have been obtained from the open circuit voltage, short circuit current density, and the maximum points of voltage and current density generated from the cell.

**Specifications Table**

**Table:** 

Subject area	*Applied physics*
More specific subject area	*Solar energy*
Type of data	*Table and figure*
How data was acquired	*The values of the materials have been accumulated from the references*[1], [2], [3], [4], [5], [6], [7], [8], [9], [10], [11], [12]*. Afterwards, the numerical simulation was conducted by ADEPT 2.1 simulation tool*[13]*using the layer data and thus the output parameters of the CIGS solar cell have been filtered.*
Data format	*Filtered and analyzed*
Experimental features	*The solar cell has been designed as SLG/Mo/CIGS/ZnS/i-ZnO/ZnO/Al-grid stack. Thereafter, the impacts of different variable parameters of the constituent materials have been taken into consideration. Finally, the cell performance has been computed from the simulation study.*
Data accessibility	*Data is in the article*

**Value of the data**

• This dataset can be used to compare the theoretical result of other CIGS solar cell models.
• Using these dataset researchers can easily develop a theoretical model of a solar cell.
• The simulation approach used in other simulators can be justified by using the simulated performance data.
• These data will be helpful to expand the idea of numerical modeling before fabrication of CIGS solar cells.

## 1 Data

**Fig. 1:**
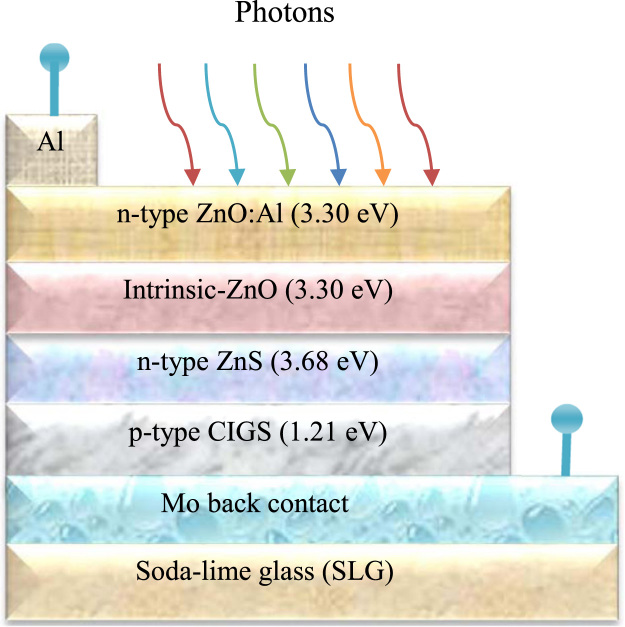
Schematic diagram of CIGS photovoltaic solar cell.

**Fig. 2:**
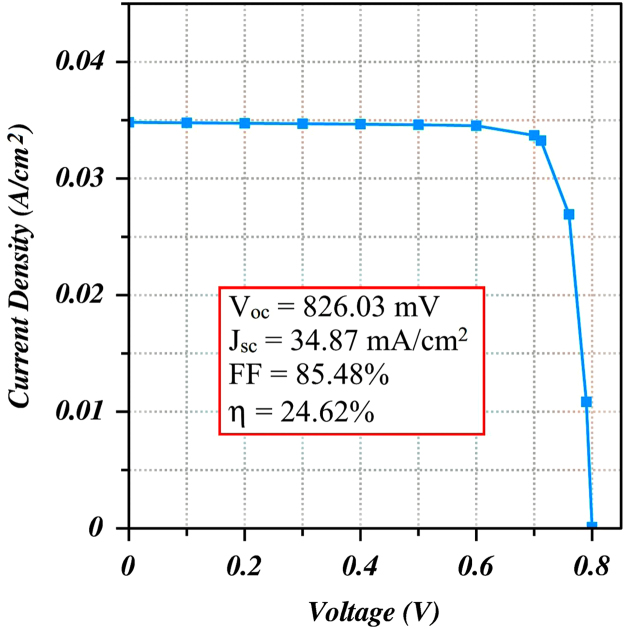
J-V characteristic curve for optimized CIGS solar cell.

The values of the materials which have been used different layers for simulation to design a CIGS solar cell are presented in Table 1. The reflectance and the recombination velocity for holes and electrons for both front and back contact layer of the cell have been listed in Table 2. The simulation was carried out under some simulation conditions that have been represented by Table 3. All of these data has been assumed from the published research articles [1], [2], [3], [4], [5], [6], [7], [8], [9], [10], [11], [12]. Fig. 1 lays out the schematic design for ZnO:Al/i-ZnO/ZnS/CIGS structure. Consequently, Fig. 2 shows the J-V characteristic curve for optimized CIGS photovoltaic cell whereas Table 4 reports the performance parameters of the cell.

**Table 1:** Input parameters for CIGS solar cell simulation with ZnS buffer.

Parameters	n-ZnO: Al	i-ZnO	n-ZnS	p-CIGS
Thickness, $t_{m}\left(\mathrm{\mu m}\right)$	0.20	0.02	0.04	3.00
Dielectric constant, $K_{s}$	7.80	7.80	8.28	13.60
Refractive index, $N_{\mathrm{dx}}$	2.00	2.00	3.16	3.67
Band gap, $E_{g}\left(\mathrm{eV}\right)$	3.30	3.30	3.68	1.21
Electron affinity, $\chi_{e}\left(\mathrm{eV}\right)$	4.60	4.60	4.13	4.21
Electron mobility, $\mu_{n}({\mathrm{cm}}^{2}V^{-1}s^{-1})$	160	130	250	100
Hole mobility, $\mu_{p}({\mathrm{cm}}^{2}V^{-1}s^{-1})$	40	30	70	25
Conduction band effective density of states, $N_{c}({\mathrm{cm}}^{-3})$	2.2×10^18^	1.5×10^18^	1.7×10^18^	2×10^18^
Valence band effective density of states, $N_{v}({\mathrm{cm}}^{-3})$	1.8×10^19^	1.6×10^19^	2.4×10^19^	1.6×10^19^
Donor concentration, $N_{d}({\mathrm{cm}}^{-3})$	1×10^18^	–	5×10^16^	–
Acceptor concentration, $N_{a}({\mathrm{cm}}^{-3})$	–	–	–	3×10^16^
Electron lifetime, $\tau_{n}\left(s\right)$	5×10^-8^	3×10^-8^	2×10^-8^	1×10^-8^
Hole lifetime, $\tau_{p}\left(s\right)$	5×10^-9^	3×10^-9^	6×10^-8^	5×10^-8^

**Table 2:** Contact layer data of CIGS solar cell simulation.

Parameters	Front contact	Back contact
Reflectance, R_f_	0.2	0.8
Barrier height, Φ_b_	0.03	1.90
Recombination velocity for holes, S_p_	10^7^	10^7^
Recombination velocity for electrons, S_n_	10^7^	10^7^

**Table 3:** Operating conditions for the simulation of CIGS solar cell.

Operating conditions	Value
Standard illumination spectra	AM1.5 G
Solar input power, $E({\mathrm{Wm}}^{-2})$	1000
Temperature, $T_{c}\left(\mathrm{{}^{\circ}C}\right)$	27
Shadowing factor (%)	10

**Table 4:** Performance parameters of optimized CIGS cell vs. reference cell [14].

Performance parameters	Experimental reference cell	Optimum simulated result
$V_{\mathrm{oc}}\left(\mathrm{mV}\right)$	671.00	826.03
$J_{\mathrm{sc}}({\mathrm{mAcm}}^{-2})$	34.90	34.87
FF(%)	77.60	85.48
η(%)	18.10	24.62

## 2 Experimental design, materials and methods

### 2.1 Device structure

The schematic diagram for CIGS photovoltaic solar cell has been shown in Fig. 1. The structure forms a stack of materials Mo/Cu(In_,_Ga)Se_2_/ZnS/i-ZnO/ZnO:Al/Al-grid for modeling of a CIGS cell. Soda lime glass (SLG) with Mo has been used as a back contact.

### 2.2 Performance analysis

ADEPT 2.1 is a one-dimensional online simulator which has been used to analyze the electrical and optical characteristics and consequently the performance of the proposed CIGS solar cell. From the J-V characteristic curve shown in Fig. 2, the performance parameters such as open-circuit voltage (V_oc_), short-circuit current density (J_sc_), fill factor (FF), and efficiency (η) are computed after conducting the simulation. In Table 4, the performance parameters of the CIGS solar cell have been reported and compared with an experimental reference cell.
